# Colonization Dynamics of Cefotaxime Resistant Bacteria in Beef Cattle Raised Without Cephalosporin Antibiotics

**DOI:** 10.3389/fmicb.2018.00500

**Published:** 2018-03-21

**Authors:** Raies A. Mir, Thomas A. Weppelmann, Lin Teng, Alexander Kirpich, Mauricio A. Elzo, Joseph D. Driver, Kwangcheol C. Jeong

**Affiliations:** ^1^Emerging Pathogens Institute, University of Florida, Gainesville, FL, United States; ^2^Department of Animal Sciences, Institute of Food and Agricultural Sciences, University of Florida, Gainesville, FL, United States; ^3^Herbert Wertheim College of Medicine, Florida International University, Miami, FL, United States; ^4^Department of Molecular Genetics and Microbiology, College of Medicine, University of Florida, Gainesville, FL, United States

**Keywords:** antimicrobial resistance, ESBL, cefotaxime resistance, cattle, metagenomic analysis

## Abstract

The emergence of infections caused by antimicrobial resistant microorganisms (ARMs) is currently one of the most important challenges to public health and medicine. Though speculated to originate at least partially from the overuse of antibiotics during food animal production, we hypothesized that cattle are exposed to ARMs in the environment. In this cohort study, a herd of beef calves with no previous exposure to antibiotics was followed during the first year of life in order to investigate the rate of colonization by bacteria resistant to the third-generation cephalosporin cefotaxime. Fecal samples were collected from the recto anal junction of cattle at the age of ~3, 6, 9, and 12 months and tested for cefotaxime resistant bacteria (CRB) and the presence of extended spectrum β-lactamases (ESBLs). The colonization dynamics of CRB in calves (*n* = 188) was evaluated with samples collected from four periods using longitudinal statistical analyses. Colonization by CRB was a dynamic process with over 92% of the calves testing positive for CRB at least once during the first year of life. All isolates subjected to antimicrobial susceptibility test were resistant to at least four different antibiotics and carried multiple variants of the *bla*CTX-M genes. Metagenomic analysis revealed significant differences in microbiota of the calves with and without CRB colonization at different ages. This study provides evidence that colonization of beef calves by ARMs is a dynamic process that can occur in the absence of veterinary or agricultural use of antibiotics.

## Introduction

More than 23,000 deaths have been attributed to infections from antimicrobial resistant microorganisms (ARMs) in the United States annually; with infections resistant to antibiotics reported from hospitals in more than 100 countries around the world (WHO, 2014). The acquisition of antimicrobial resistance by pathogens has challenged our ability to treat infections and the emergence of ARMs represents a serious contemporary threat to public health and medicine (Ventola, 2015). Exposure of bacteria to antimicrobial compounds leads to the survival and proliferation of only those that adapted to survive in the presence of such compounds (Capita et al., 2014). This process, termed positive selection, often involves the acquisition of genetic elements that are integrated into the genome of the organism or contained on a plasmid inside the bacteria (Roe and Pillai, 2003). Since the development of antimicrobial resistance involves selection pressure, environments with frequent use of antibiotic compounds such as hospitals, communities, and food animal production are the most likely source of ARMs (Landers et al., 2012).

Once commensal bacteria in food animals transfer acquired antibiotic resistance to pathogenic bacteria, transmission to humans can result in infections that are highly resistant to therapy with one or multiple antibiotics (Guillemot and Courvalin, 2001). The positive correlation between the prevalence of ARMs in food animals and ARMs in humans (Vieira et al., 2011) has led to the hypothesis that the overuse of antibiotics during food production is partially responsible for the spread of ARMs throughout communities and hospitals (Landers et al., 2012). The third-generation cephalosporin, cefotaxime, is used widely in human medicine for the treatment of bacterial infections including potentially life-threatening meningitis and is on the World Health Organizations list of essential medicines (Sumano et al., 2004; FDA, 2012). Bacteria become resistant to cephalosporins by the production of β-lactamase enzymes including extended spectrum β-lactamases (ESBLs) (Rubin and Pitout, 2014).

In the previous study (Mir et al., 2016b), we have reported the isolation of bacteria resistant to cefotaxime (CRB), a third-generation cephalosporin, in cattle with no previous exposure to antibiotics. In that pilot study, only a single sample was taken from each animal, leaving no way to measure the rates of CRB colonization over time, the duration of CRB colonization, or if previously colonized animals can become uncolonized. Thus, the objective of the current study was to determine the prevalence and dynamics of CRB in a cohort of beef cattle without previous exposure to prophylactic or therapeutic antibiotics. By using culture-based isolation techniques and molecular genetics, we were also able to determine whether CRBs were resistant to multiple antibiotics, investigated the genes responsible for ESBL activity, and identified CRB at the species level. Additionally, metagenomic analysis was used to assess the relationships between the diversity of microorganisms or abundance of individual microorganisms in the gastrointestinal tract and CRB colonization in cattle.

## Materials and methods

### Ethics statement

Standard practices of animal care and use were applied to animals used in this project. The research protocols used in this study were approved by the University of Florida Institutional Animal Care and Use Committee (IACUC Protocol #: 201408629).

### Animal management and sample collection

All calves were born and raised at the Beef Research Unit of University of Florida (Gainesville, Florida, USA in North Central Florida) with no exposure to sub-therapeutic antimicrobials. The herd of beef calves consisted of a multi-breed population derived from Brahman and Angus cattle managed under a loose system of housing with average stocking density equal to 1.5 acres (0.6 Ha) per animal. Calves were tagged with a unique identification number and followed from birth throughout their first year of life with samples collected four times approximately every 3 months. The sample period, month of collection, approximate age of the calves, and the numbers of fecal samples collected were as follows: Sample 1 March, calves 0 to 3 months (*n* = 259); Sample 2 June, calves 3 to 6 months (*n* = 263); Sample 3 August, calves 6 to 9 months (*n* = 261); Sample 4 December, calves 9 to 12 months (*n* = 193). Around 70 calves had been sold or moved to a different farm, so December sampling had only 193 samples. The sampling scheme resulted in 188 animals with fecal swabs collected during all four sampling time points. Fecal samples were collected from the recto-anal junction (RAJ) of calves using sterile cotton swabs (Fisher Scientific, USA) and placed in sterile 15 mL conical tubes (Falcon, USA). All samples were transported on ice and processed the same day using protocol described in the following section.

### Identification of cefotaxime resistant bacteria

A combination of culture-based and nucleic acid-based methods were used for the detection of cefotaxime resistant bacteria (CRB) from the fecal samples. Fecal swabs were suspended with 2 mL of tryptic soy broth (TSB), spread plated onto MacConkey agar containing 4 μg/mL cefotaxime (BD, USA) at various dilutions in TSB (10^0^ to 10^−2^), and incubated overnight at 37°C. After 24 h, bacterial colonies were enumerated prior to the purification of up to six colonies on MacConkey agar containing 4 μg/mL cefotaxime and stored at −20°C in 15% glycerol for further characterization.

### Analyses of CRB colonization dynamics

**Figure 1:**
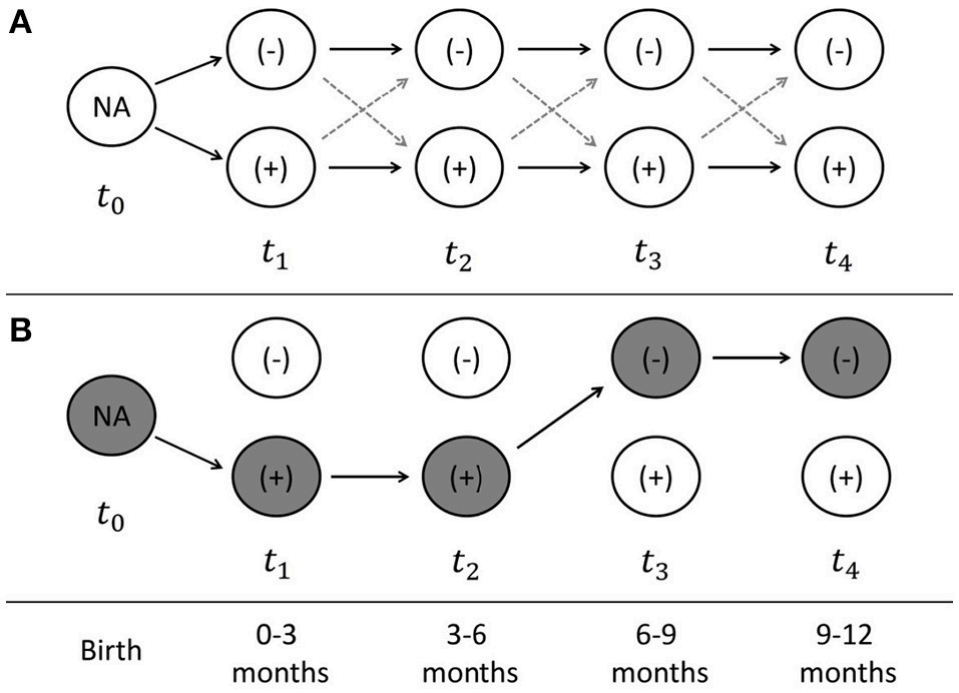
Sampling scheme and model of longitudinal probability of colonization. The sampling scheme used for the longitudinal analyses of calves with four consecutive time points is presented **(A)**. At the time of birth (t0), the colonization by antimicrobial resistant microorganisms (ARMs) is unknown, after which time each cattle can become colonized or uncolonized at times (t_1_–t_4_), which correspond to the time periods from 0–3, 3–6, 6–9, and 9–12 months of age. An example of the state of colonization is presented **(B)** for a calve that becomes colonized at 0–3 months, remains colonized at 3–6 months, before becoming uncolonized at 6–9 months, and remaining uncolonized at 9–12 months. The probability of colonization at each time point can be directly calculated, as well as the string of probabilities that each calve can take from birth to the end of the first year of life.

Cattle containing CRB in the gastrointestinal tract were classified as CRB positive and the longitudinal trends in colonization were analyzed based on the presence and concentration of CRB. Simple logistic regression was used to determine the association between colonization by CRB and the calf breed group (Brahman, Angus, or “Brangus” hybrid) and the sex of the calf (bull or heifer) at any single point in time. For the animals with four consecutive samples collected (*n* = 188), the marginal increase or decrease in the proportion of calves colonized by CRB between each sample was investigated using McNemar's test for matched pairs. Cattle were assumed to be born without CRB colonization and classified at each sampling into one of four groups: calves not previously colonized that remain uncolonized (−/−), calves not previously colonized that became colonized (−/+), calves previously colonized that remain colonized (+/+), and calves previously colonized that became uncolonized (+/−). Using Markov chain assumptions, the probability of colonization between sampling periods was assumed to only be dependent on the previous state of colonization and calculated directly (Figure 1). All statistical analyses were conducted using STATA software package (STATA® MP 11.2, StataCorp, USA) with a significance threshold of α = 0.05.

### PCR detection of ESBL encoding genes from CRB

Frozen cefotaxime resistant isolates (*n* = 2,689) were revived on MacConkey agar plates containing 4 μg/mL cefotaxime and screened by polymerase chain reaction (PCR) for the presence of the *bla*CTX-M gene using primers, KCP 685 and KCP 686 (Table 1) as described previously (Edelstein et al., 2003). The amplicons (~544 bp) from 60 *bla*CTX-M positive isolates were purified using the QIAquick PCR Purification Kit (Qiagen, USA) and sequenced using the Sanger sequencing method at the Interdisciplinary Center for Biotechnology Research at University of Florida. The sequences were aligned using ClustalW method (BioEdit®) prior to phylogenetic analysis conducted using MEGA (version 6). Briefly, sequences were analyzed for nucleotide substitution using Jukes & Cantor (JC) model with a uniform distribution. The aligned *bla*CTX-M gene sequences were used to construct a maximum likelihood phylogenetic tree with 1000 bootstrap replications to represent the genetic relatedness of *bla*CTX-M gene in CRB.

**Table 1:** Primers used in this study.

**Target genes**	**Primer name**	**Sequences**	**Size (bp)**	**References**
*bla* CTX-M-F	KCP 685	TTTGCGATGTGCAGTACCAGTAA	544	Edelstein et al., 2003
*bla* CTX-M-R	KCP 686	CGATATCGTTGGTGGTGCCATA		
16S rRNA-F	KCP 812	CAG GCC TAA CAC ATG CAA GTC	1300	Marchesi et al., 1998
16S rRNA-R	KCP 813	GGG CGG WGT GTA CAA GGC		

### Identification of bacterial isolates by 16S rRNA gene sequencing

CRB strains were revived on MacConkey agar plates containing 4 μg/mL cefotaxime before genomic DNA was extracted from 60 *bla*CTX-M positive isolates using the QIAamp DNA Mini Kit. The extracted gDNA was used to amplify the 16S rRNA gene was using primers, KCP 812 (5′- CAG GCCTAACACATGCAAGTC - 3′) and KCP 813 (5′- GGGCGGWGTGTACAAGGC - 3′) (~1300 bp) (Marchesi et al., 1998) (Table 1). The PCR products were purified using the QIAquick PCR Purification Kit and sent for Sanger sequencing. The resulting sequences were aligned with ClustalW method (BioEdit®). The aligned sequences were analyzed in MEGA (version 6) using Jukes & Cantor (JC) model with Gamma distribution. The maximum likelihood tree was constructed from the sequences using Bootstrap method with 1000 bootstrap replications.

### Antimicrobial susceptibility test

To determine if the CRB were also resistant to other antibiotics, we tested the representative 36 out of 60 isolates for resistance against 10 antibiotics that represent different classes of antibiotics in a semi-quantitative test by determining the colony size on an antibiotic containing media plate. The *bla*CTX-M positive isolates were purified and inoculated in Luria Bertani (LB) broth containing cefotaxime (4 μg/mL) to a concentration of ~1 × 10^8^ bacteria/mL (OD_600_ = 0.2–0.4) before plating on the Mueller Hinton Agar (Becton Dickinson, Franklin Lakes, NJ) following the CLSI guidelines. The following antibiotics were used Cefotaxime (4 μg/mL), Erythromycin (20 μg/mL), Nalidixic acid (30 μg/mL), Tetracycline (15 μg/mL), Ampicillin (50 μg/mL), Kanamycin (50 μg/mL), Potassium Tellurite (2.5 μg/mL), Rifampicin (100 μg/mL), Chloramphenicol (35 μg/mL), and Polymixin B (50 μg/mL). The plates were incubated at 37°C and the isolates were classified as negative (−), mild resistant (+), and highly resistant (++) based on the diameter of the growth on the antibiotic containing media after overnight incubation (no growth, <2 mm grown, and ≥ 2 mm grown, respectively).

To further characterize the antimicrobial resistance of these CRB and generate their antibiogram, 36 isolates were selected and subjected for antimicrobial susceptibility test (AST) against 12 antibiotics according to the guidelines of the Clinical and Laboratory Standards Institute (CLSI, 2015). Standard Kirby Bauer disk diffusion method was used on Mueller Hinton agar to generate antibiogram. The antibiotics disks included Amikacin (30 μg), Ampicillin (10 μg), Amoxycillin/Clavulanic acid (30 μg), Sulfisoxazole (0.25 mg), Ceftiofur (30 μg), Chloramphenicol (30 μg), Cephalothin (30 μg), Gentamicin (10 μg), Nalidixic acid (30 μg), Streptomycin (10 μg), Sulfamethoxazole/trimethoprim (23.75 μg/1.25 μg), and Tetracycline (30 μg). The control strains in this assay were *Escherichia coli* (ATCC 25922), *Staphylococcus aureus* (ATCC 25923), and *Pseudomonas aeruginosa* (ATCC 27853).

### Metagenomic analysis

To determine the abundance of microbiota and its association with cefotaxime resistance (CefR), a metagenomic analysis of the fecal samples from the calves was conducted using 454 pyrosequencing (Macrogen Inc., South Korea). A total of 48 samples, collected at four different sampling points from 12 animals that included both cefotaxime resistant (CefR, *n* = 24) and cefotaxime susceptible samples (CefS, *n* = 24), were used to compare the taxonomic profile and microbial diversity. We also collected 10 soil samples each in September and December from the same farm where animals were grazing to understand the role of soil microbiota in CRB dynamics. The DNA was extracted from the fecal and soil samples using the PowerSoil DNA Isolation kit (MoBio Laboratories, USA) according to the manufacturer's protocol. A gDNA library was prepared using PCR products according to the GS FLX plus library prep guide. Libraries were quantified using Picogreen assay (Victor 3). The emPCR, corresponding to clonal amplification of the purified library, was carried out using the GS-FLX plus emPCR Kit (454 Life Sciences). Briefly, libraries were immobilized onto DNA capture beads. The library-beads obtained were added to a mixture of amplification mix and oil and vigorously shaken on a Tissue Lyser II (Qiagen) to create “micro-reactors” containing both amplification mix and a single bead. Emulsion was dispensed into a 96-well plate and the PCR amplification program was run according to the manufacturer's recommendations. Twenty monogram aliquot of each sample DNA was used for a 50 μL PCR reaction. The 16S universal primers 27F (5′-GAGTTTGATCMTGGCTCAG-3′), 518R (5′-WTTACCGCGGCTGCTGG-3′) were used for amplifying of 16S rRNA genes. FastStart High Fidelity PCR System (Roche) was used for PCR under the following conditions: 94°C for 3 min followed by 35 cycles of 94°C for 15 s; 55°C for 45 s and 72°C for 1 min; and a final elongation step at 72°C for 8 min. After the PCR reaction, the products were purified using AMPure beads (Beckman coulter, USA) and sequenced using the following method by Macrogen Ltd. (Seoul, Korea). Following PCR amplification, the emulsion was chemically broken and the beads carrying the amplified DNA library were recovered and washed by filtration. Positive beads were purified using the biotinylated primer A (complementary to adaptor A), which binds to streptavidin-coated magnetic beads. The DNA library beads were then separated from the magnetic beads by melting the double-stranded amplification products, leaving a population of bead-bound single-stranded template DNA fragments. The sequencing primer was then annealed to the amplified single-stranded DNA. Lastly, beads carrying amplified single-stranded DNA were counted with a Particle Counter (Beckman Coulter, USA). Sequencing was performed on a Genome Sequencer FLX plus (454 Life Sciences), and each sample was loaded in 1 region of a 70–75 mm PicoTiter plate (454 Life Sciences) fitted with a 8-lane gasket.

### Selection of 16S rRNAs and taxonomic assignment

Using the basic local alignment search tool (BLAST), all the sequence reads were compared to Silva rRNA database. Sequence reads which had sequence similarity with less than 0.01 E-value were admitted as partial 16S rRNA sequences. Non-16S rRNA sequence reads comprised less than 1% of all reads. Taxonomic assignment of the sequenced read was carried out using NCBI Taxonomy Databases. The five most similar sequences for each sequence read were found by their bit scores and E-value from BLAST program. Needleman-Wunsch global alignment algorithm was used to find the optimum alignment of two sequences along their entire length. A pairwise global alignment was performed on selected candidate hits to identify the best aligned hit. The taxonomy of the sequence with the highest similarity was assigned to the sequence read. By the similarity, we assigned the taxonomy down to these taxonomical hierarchies; species with more than 97% similarity, genus 94%, family 90%, order 85%, class 80%, and phylum 75%.

### Operational taxonomic unit (OTU) analysis for community richness

CD-HIT-OTU software was used for clustering. Mothur software was used for analyzing microbial communities and Shannon-Weaver diversity index and Simpson index were used for species diversity. Statistical analysis of the metagenomic data for the microbiota diversity and richness within the calves for different ages was analyzed using SAS (Version 9.4) and R program (R Development Core Team) was used to generate heat maps for the various OTUs present in our fecal samples. Mean proportions and 95% CIs were used to describe the changes in proportions of the 16S rRNA reads assigned to different OTUs present in the fecal samples. Mean proportions of the bacterial taxa (OTUs) within the study groups were analyzed using a generalized linear mixed model (GLIMMIX) in SAS (version 9.4, SAS Institute Inc., USA). The heat maps were generated by the free online G-plot package in R-program.

## Results

### Prevalence and concentration of cefotaxime resistant isolates

**Figure 2:**
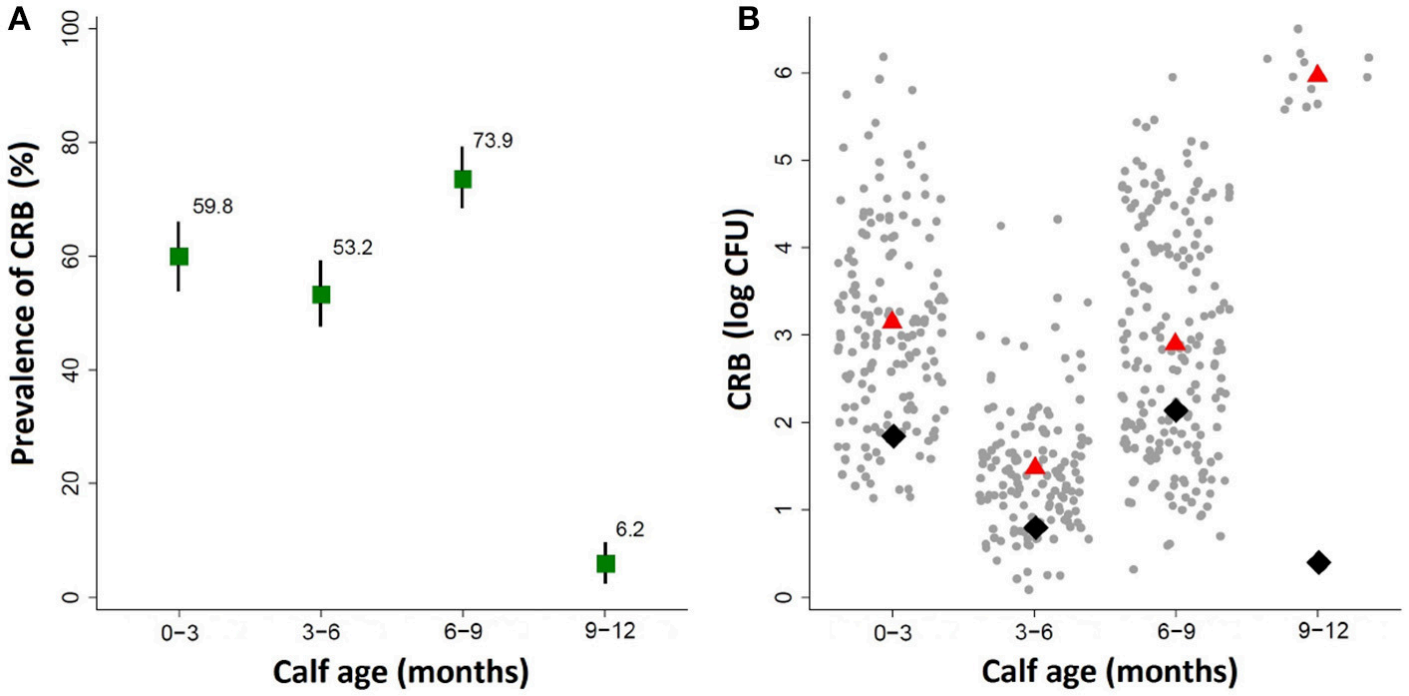
Prevalence of ARM colonization during the first year of life. The prevalence and concentration of CRB isolated from fecal swabs collected at four intervals during the first year of life in a cohort of beef calves are presented. **(A)** The prevalence of cattle colonized by ARMs and the corresponding 95% confidence intervals are shown in the top panel. **(B)** The average concentration of all animals (black diamond), and the average concentration of only animals that were colonized (red triangle), along with a scatter plot of the concentrations (log CFU) from each sample (gray dots).

By the first sample collection from calves aged 0–3 months, the majority of the calves had already become colonized by cefotaxime resistant bacteria (CRB) (Figure 2A). The prevalence of CRB collected from calves that were 0–3 and 3–6 months old were 59.8% (95% CI: 53.8, 65.9) and 53.2% (95% CI: 47.2, 59.3), respectively. The prevalence in the herd reached a peak at the age of 6–9 months where 73.9% (95% CI: 68.6, 79.3) of the calves were colonized by CRB, before decreasing at the age of 9–12 months to only 6.2% (95% CI: 2.8, 9.7). The likelihood of colonization by CRB in calves sampled during the first year of life was significantly different by the age of the calves (*P* < 0.001). Compared to calves aged 0–3 months, the prevalence of CRB in calves aged 3–6 months was not significantly different, however calves aged 6–9 months had a significantly higher (*P* = 0.001) likelihood of being colonized (OR: 1.90; 95% CI: 1.31, 2.76) and calves aged 9–12 months had a significantly lower (*P* < 0.001) likelihood of being colonized (OR: 0.04; 95% CI OR: 0.24, 0.84). The average concentrations of CRB from samples collected during the first year of life are presented in Figure 2B along with the average concentration for all calves (black diamond) and only those that were colonized (red triangle). The average concentrations and interquartile ranges for cattle colonized by CRB expressed as (log_10_ CFU) were as follows: 3.11 (IQR: 2.17, 3.79) at age 0–3 months, 1.47 (IQR: 1.00, 1.83) at age 3–6 months, 2.86 (IQR: 1.78, 4.00) at age 6–9, and 5.99 (IQR 5.85, 6.12) at age 9–12 months. The concentration of CRB in calves that were colonized was also significantly associated with the age of the calf (*P* < 0.001). Compared to calves aged 0–3 months, calves aged 3–6 months had a significantly lower (*P* < 0.001) concentration of CRB (OR = 0.19; 95% CI: 0.15, 0.25), calves aged 6–9 months has only a slightly lower (*P* = 0.026) concentration of CRB (OR = 0.78; 95% CI: 0.62, 0.97), and calves aged 9–12 months had a significantly higher (*P* < 0.001) CRB (OR = 17.7; 95% CI: 19.05, 26.56). Notably, though only twelve calves were colonized during the 9–12 months' sample, all were shedding between10^5^ and 10^6^ CFU of CRB.

### Characterization of a subsample of CRB

**Figure 3:**
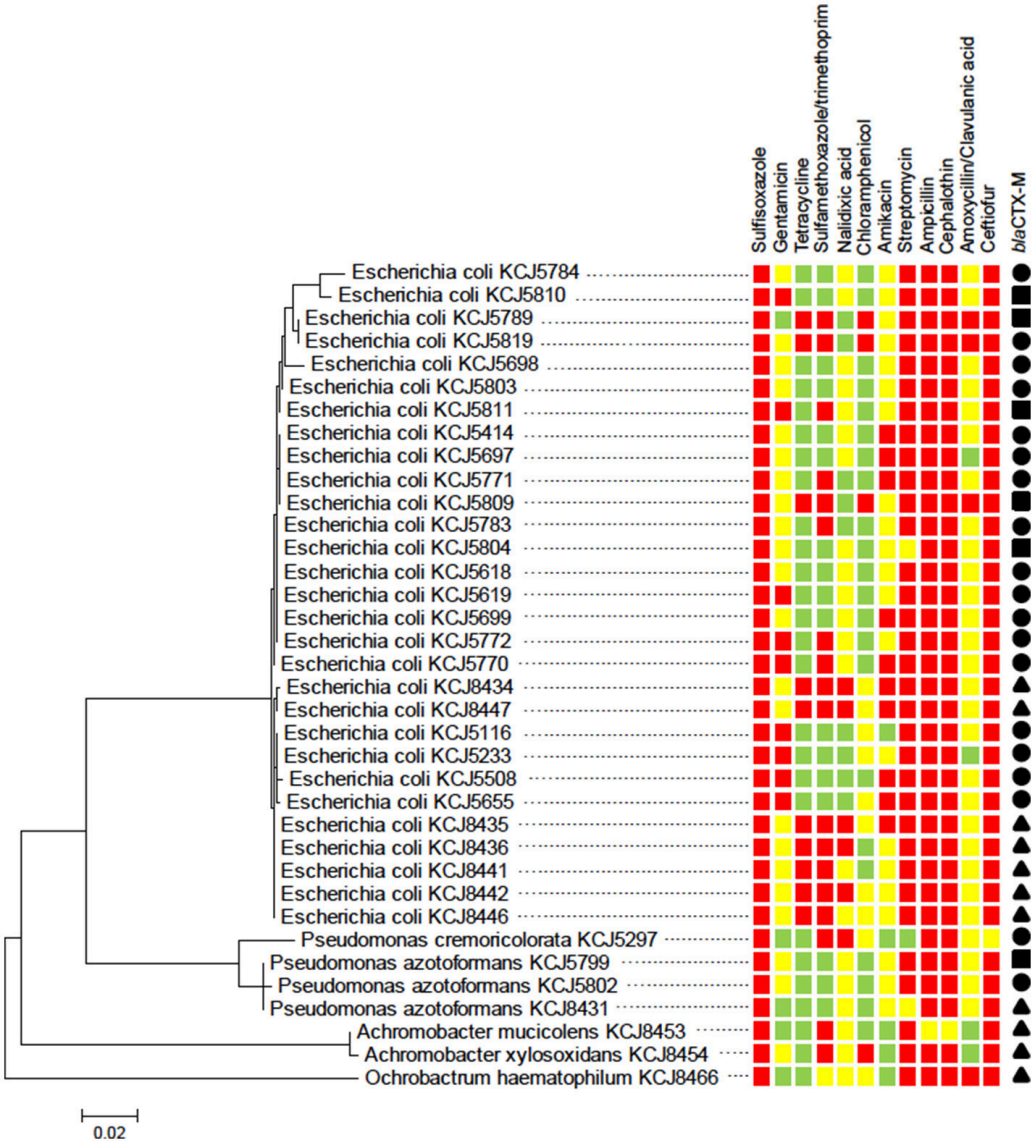
Cefotaxime resistant isolates from calves show multidrug resistance. The phylogenetic relatedness of 16S rRNA gene of 36 isolates was analyzed by using the Maximum Likelihood method based on the Jukes-Cantor model. The isolates were subjected to antimicrobial susceptibility test (AST) to investigate their multi-drug resistance phenotype. All of the isolates showed resistance to three antibiotics at least: susceptible (green squares), intermediate resistance (yellow squares), and resistance (red squares). *bla*CTX-M type: CTX-M-1 (solid circle), CTX-M-15 (solid triangle), and CTX-M-32 (solid square).

The susceptibility of the isolates to a range of different antimicrobial compounds representing different classes of antibiotics is presented (Figure S1 and Figure 3). All of the isolates (*n* = 60) were resistant to at least five or more antibiotics (multi-drug resistant); with 18, 45, 35, and 2% of the isolates resistant to five, six, seven, and eight antibiotics, respectively. All isolates were highly resistant to the beta-lactams (cefotaxime and ampicillin), mildly resistant to erythromycin, and susceptible to the synthetic compounds potassium tellurite and nalidixic acid. The percentage of the isolates with no, mild, or high resistance toward the other antibiotic compounds tested were as follows: 35% none, 28.3% mild, and 36.7% high toward polymyxin B; 10% none, 78.3% mild, and 11.7% high toward tetracycline; 3.3% none and 96.7% mild toward kanamycin; 35% none and 65% mild toward rifampicin; and 96.7% none, 1.7% mild, and 1.7% high toward chloramphenicol. The identification of CRB as determined by 16S rRNA sequencing at the genus and species level is presented (Figure 3). The majority of the isolates were *Escherichia coli* followed by *Pseudomonas* spp., *Achromobacter* spp., and *Ochrobactrum* spp. CRB isolates were further evaluated for multi-drug resistance against 12 different antibiotics according to CLSI guidelines (CLSI, 2015). As shown in Figure 3, all of the isolates were resistant to at least 3 antibiotics. Notably, three isolates (*E. coli* KCJ8434, KCJ8435, and KCJ8447) were resistant to 9 antibiotics, except amoxycillin/clavulanic acid, gentamycin, and chloramphenicol. All *E. coli* isolates were resistant to sulfisoxazole, ampicillin, cephalothin, and ceftiofur, and compared with the other species, *E. coli* showed wide range of resistance against 5 to 9 different antibiotics. Three variants of *bla*CTX-M gene were identified in the isolates: *bla*CTX-M-1, *bla*CTX-M-15 and *bla*CTX-M-32 (Figure 3). Isolates from calves aged 0–3 months predominantly contained the *bla*CTX-M-1 (84%) and *bla*CTX-M-32 (16%) genotypes, while the isolates from calves aged 6–9 months only contained the *bla* CTX-M-15 (100%) genotype.

### Dynamics of calf colonization by antimicrobial resistant microorganisms

**Figure 4:**
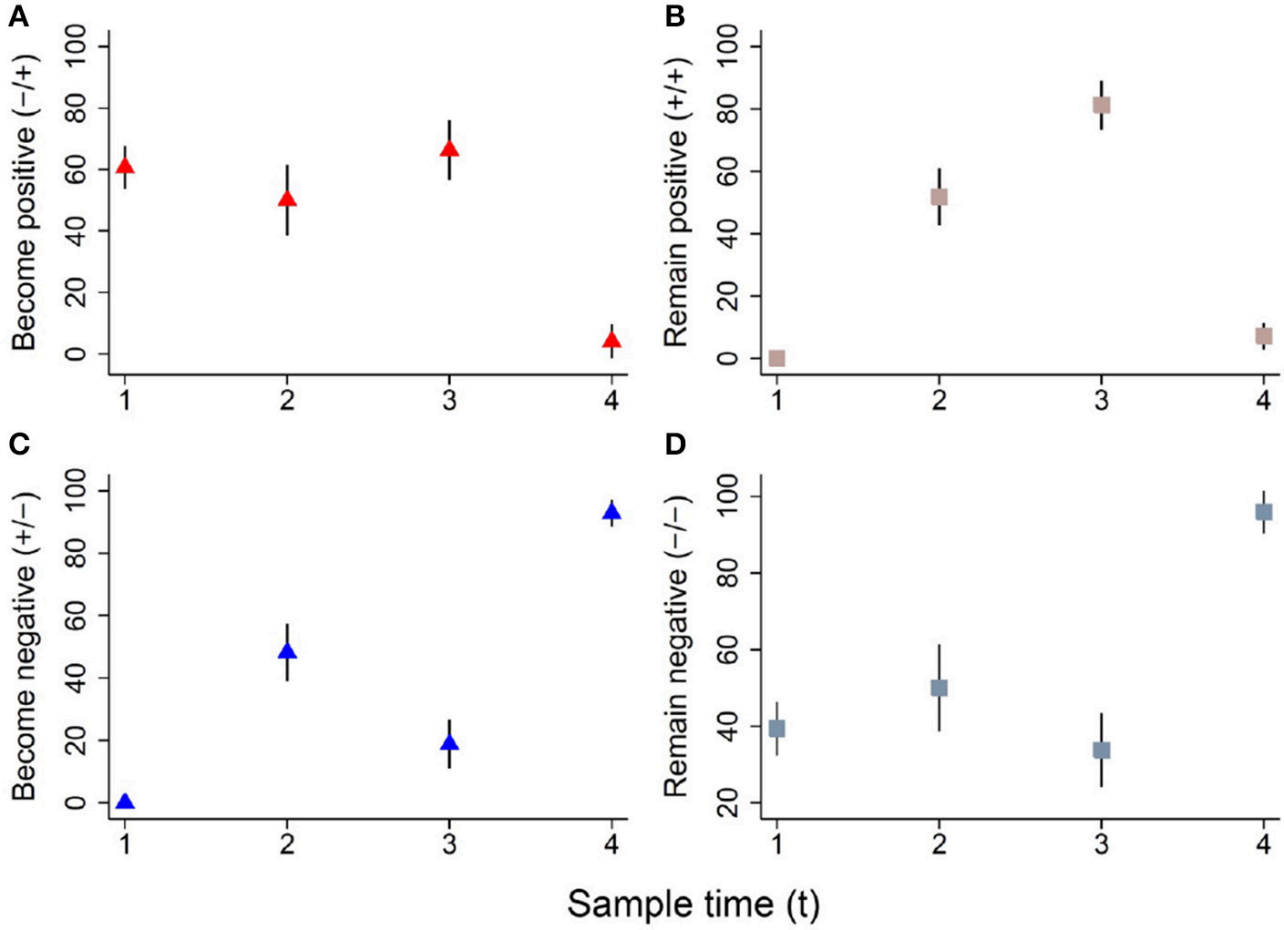
Dynamics of ARM colonization during the first year of life. The colonization dynamics of beef calves (*n* = 188) with four samples collected during the first year of life are presented. The proportion (%) of animals that became colonized, remained colonized, became uncolonized, or remained uncolonized between sampling times (t_x_ to t_x+1_) are shown in panels **(A–D)**, respectively. The following colonization dynamics are presented for cattle that became positive (**A**, red triangle), remained positive (**B**, pink square), became negative (**C**, blue triangle), and remained negative (**D**, gray square).

**Figure 5:**
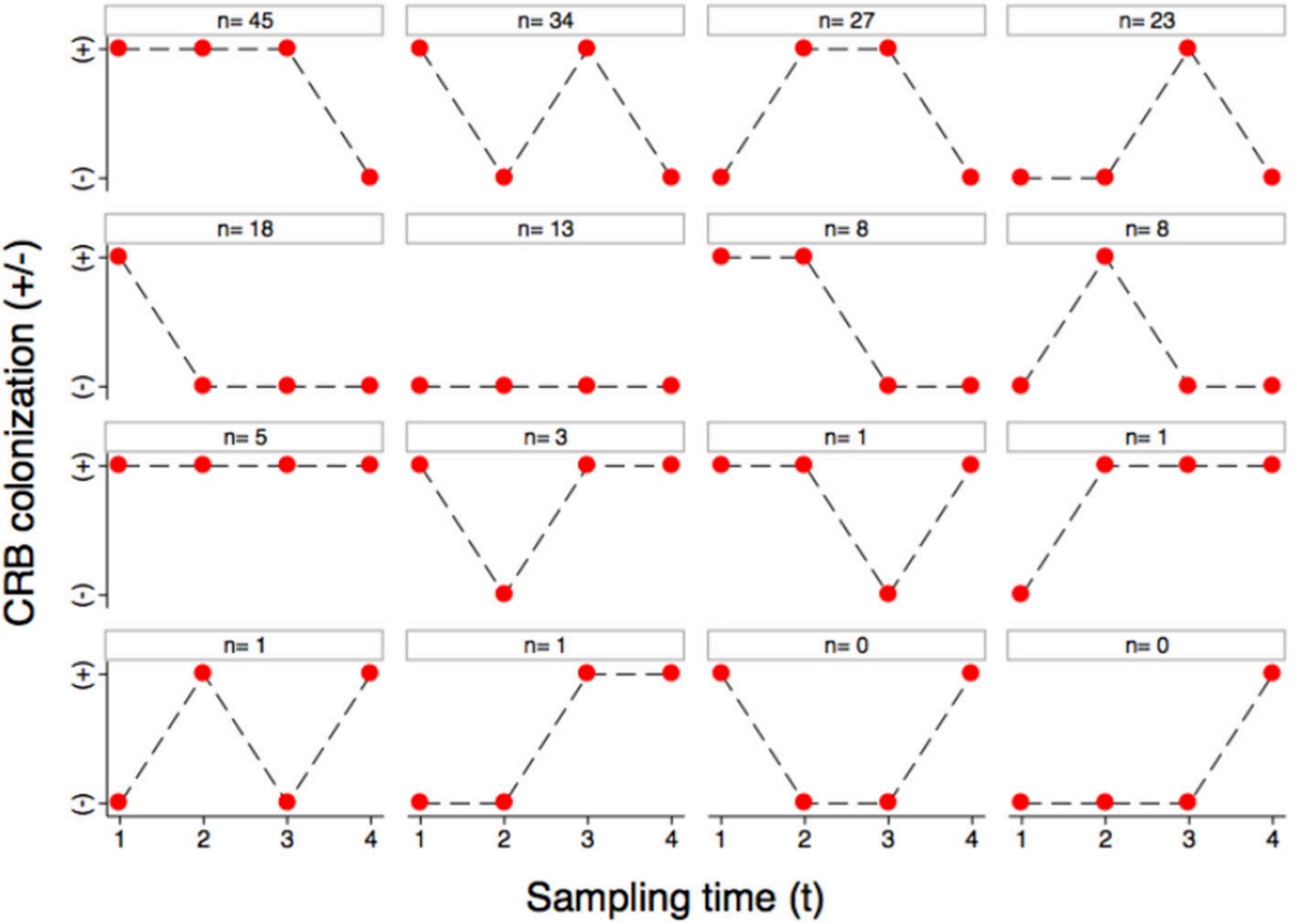
Longitudinal colonization dynamics. The colonization dynamics of beef calves (*n* = 188) with four samples collected during the first year of life are presented. All of the animals that were colonized (+) or not colonized (−) at the four sample collections (1–4) are shown with respect to the 16 possible strings of colonization states with the number of animals observed to follow that string.

The colonization dynamics by CRB during the first year of life for a subset of calves (*n* = 188) with matched samples from four time points were analyzed. The transition of calves between colonization states during the first year of life is presented (Figure 4). By the first sample at 0–3 months-of-age (*n* = 188), 60.6% of the calves had become colonized by CRB, with 39.4% remaining uncolonized. Of calves that were colonized by 0–3 months-of-age (*n* = 114), 51.7% remained colonized and 48.2% became uncolonized during the second sample at 3–6 months-of-age. Of calves that were not colonized at 0–3 months-of-age (*n* = 74), 50% became colonized and 50% remained uncolonized by the second sample at 3–6 months-of-age. Of calves that were colonized at 3–6 months-of-age (*n* = 96), 81.3% remained colonized and 18.8% became uncolonized during the third sample at 6–9 months-of-age. Of calves that were not colonized at 3–6 months-of-age (*n* = 92), 66.3% became colonized and 33.7% remained uncolonized by the third sample at 6–9 months-of-age. Of calves that were colonized at 6–9 months-of-age (*n* = 139), 7.2% remained colonized and 92.8% became uncolonized during the fourth sample at 9–12 months-of-age. Of calves that were not colonized at 6–9 months-of-age (*n* = 49), 4.1% became colonized and 96.0% remained uncolonized by the fourth sample at 9–12 months-of-age. Additionally, all of the possible colonization states for calves are shown with respect to the sampling period in Figure 5. During the first year of life, over 92% of the calves were colonized by CRB, with 25.9% colonized at one sampling period, 37.6% during two sample periods, 27.0% during three sampling periods, and 2.7% during all four sampling periods.

### Relationship between intestinal microbiota and antimicrobial resistance

To investigate changes in the intestinal microbiota at different time points and the association with the presence of CRB in beef calves, the fecal microbiome from a subset (*n* = 48) of calves was analyzed using metagenomic analysis. During the first year of life, calves colonized by CRB had a higher abundance of Fusobacteria (*P* = 0.041), Elusimicrobia (*P* = 0.0025), Chlamydia (*P* = 0.047), and Cyanobacteria (*P* = 0.0391) and a lower abundance of Spirochetes (*P* = 0.022), compared to calves not colonized by CRB (Table 2).

**Table 2:** Difference in microbiota between resistant and susceptible samples^a^.

**CefR**		**CefS**	
**Aboundant OTUs^b^**	**P^c^**	**Aboundant OTUs**	***P***
p- Fusobacteria	0.041	p- Spirochaetes	0.022
p- Elusimicrobia	0.0025	o- Neisseriales	0.0065
p- Chlamydiae	0.0474	o- Aeromonadales	0.0193
p- Cyanobacteria	0.0391	o- Bacteroidales	0.0759
c- Fusobacteria	0.0483	f- Neisseriaceae	0.0401
c- Elusimicrobia	0.0027	f- Dermatophilaceae	0.0191
c- Chlamydiae	0.0474	f- Cellulomonadaceae	0.0099
o- Fusibacteriales	0.0483	f- Succinivibrionaceae	0.0193
o- Elusimicrobiales	0.0027	f- Rikenellaceae	0.0472
o- Chlamydiales	0.0474	g- Xylanibacter	0.0489
o- Rhizobiales	0.0474	g- Treponema	0.0248
o- Thermoanaerobacteriales	0.0474	g- Sarcina	0.0396

a *Mean proportions of the bacterial taxa (OTUs) within the fecal samples of calves*.

b *OTUs, Operational Taxonomical Units; p, Phylum; c, Class; o, Order; f, Family; g,Genus*.

c *Abundance of OTUs were statistically analyzed using a generalized linear mixed model in SAS, statistical significance calculated at p = 0.05*.

The following differences in the predominant commensal microbiota of the calves were observed with respect to time: increased abundance of Desulfovibrionales (*P* = 0.0132), Entomoplasmatales (*P* = 0.0072), Pasteurellales (*P* = 0.00155), Sphinobacteriales (*P* = 0.028), and Enterobacteriales (*P* = 0.0208) at age 0–3 months; Proteobacteria (*P* = 0.0003), Sphinobacteriales (*P* = 0.028), and Enterobacteriales (*P* = 0.0208) at age 3–6 months; Lachnospiraceae (*P* = 0.0064) at age 6–9 months; and Spirocahetes (*P* = 0.039) and Actinobacteria (*P* = 0.079) at age 9–12 months (Table 3).

**Table 3:** Difference in microbiota among four sampling periods^a^.

**March**		**June**		**August**		**December**	
**Aboundant OTUs^b^**	***P*^c^**	**Aboundant OTUs**	***P***	**Aboundant OTUs**	***P***	**Aboundant OTUs**	***P***
o- Desulfovibrionales	0.0132	p- Proteobacteria	0.0003	f- Lachnospiraceae	0.0064	p- Spirocahetes	0.0039
o- Entomoplasmatales	0.0072	o- Sphinobacteriales	0.028	g- Dermatophilus	0.005	p- Actinobacteria	0.0799
o- Pasteurellales	0.0155	o- Enterobacteriales	0.0208	g- Sutterella	0.0062	c- Spirochaetes	0.0039
o- Sphinobacteriales	0.028	f- Paenibacillaceae	0.0057	g- Gemella	0.0081	c- Erysipelotrichi	0.003
o- Enterobacteriales	0.0208	f- Pasteurellaceae	0.0064	g- Desulfurvibrio	0.0148	o- Spirochaetales	0.0038
f- Spiroplasmataceae	0.0089	f- Lachnospiraceae	0.0064			o- Anaeroplasmatales	0.0028
f- Desulfovibrionaceae	0.0134	g- Gemella	0.0081			o- Erysipelotrichales	0.003
f- Paenibacillaceae	0.0057	g- Paenibacillus	0.0065			f- Peptococcaceae	0.013
f- Pasteurellaceae	0.0064					f- Dermatophilaceae	0.0044
f- Lactobacillaceae	0.0002					f- Acetobacteraceae	0.0372
f- Lachnospiraceae	0.0064					f- Spirochaetaceae	0.0047
f- Rikenellaceae	0.0516					f- Anaeroplasmataceae	0.002
g- Spiroplasma	0.01					f- Peptostreptococcaceae	0.0022
g- Butyricimonas	0.0016					f- Erysipelotrichaceae	0.002
g- Desulfurvibrio	0.0148					f- Enterobacteriaceae	0.0283
g- Hespellia	0.0008					g- Xylanibacter	0.0163
g- Actinomyces	0.0153					g- Turicibacter	0.0006
g- Paraprevotella	0.0028					g- Treponema	0.0061
g- Paenibacillus	0.0065					g- Spirochaeta	0.0003
g- Blautia	0.00001					g- Ruminococcus	0.0145
g- Oscillospira	0.0096					g- Oribacterium	0.0042
g- Roseburia	0.0014					g- Dermatophilus	0.005
g- Gallibacterium	0.0189					g- Anaerosporobacter	0.0003
g- Faecalibacterium	0.0065					g- Acetobacter	0.0354
g- Lactobacillus	0.0003					g- Anaeroplasma	0.0429

a *Mean proportions of the bacterial taxa (OTUs) within the fecal samples of calves*.

b *OTUs, Operational Taxonomical Units; p, Phylum; c, Class; o, Order; f, Family; g, Genus*.

c *Abundance of OTUs were statistically analyzed using a generalized linear mixed model in SAS, statistical significance calculated at P-value = 0.05*.

**Figure 6:**
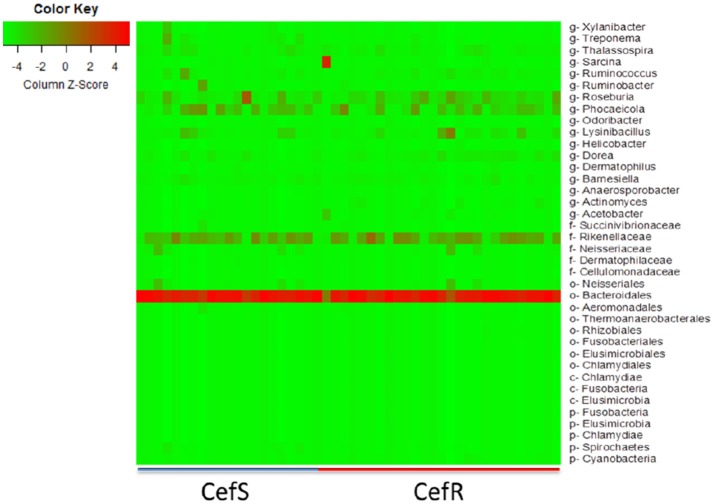
Metagenomic analysis of CefR vs. CefS calves. Microbiota of the calves was determined by the metagenomic analysis of the fecal samples. The Heat map showing the abundant OTUs in Resistant (CefR) and Susceptible samples (CefS) was generated using the R-program.

**Figure 7:**
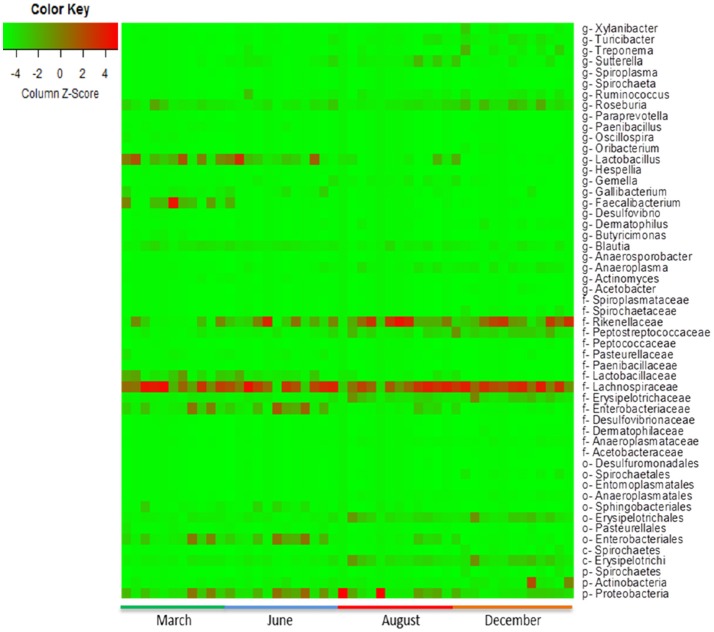
Metagenomic analysis of calves over time. The abundance of microbiota as determined by metagenomic analysis of the fecal samples from the calves collected during the first year of life is presented. The Heat map showing the abundance of OTUs in fecal samples was generated using the R-program.

The heat-map plotted using the ggplot2 package in R, graphically depicts the relative abundance of various taxa among samples collected from calves with or without colonization by CRB (Figure 6) and at four sampling periods (Figure 7). The higher abundance OTUs are depicted by red color and the OTUs, which were significantly lower in abundance among samples, are colored green. Although the causal association between a particular taxon or a group of taxa with CRB shedding by an animal is unclear, the heat-maps (Figures 6, 7) indicate that microbiota may have a role in determining resistance status of cattle.

**Figure 8:**
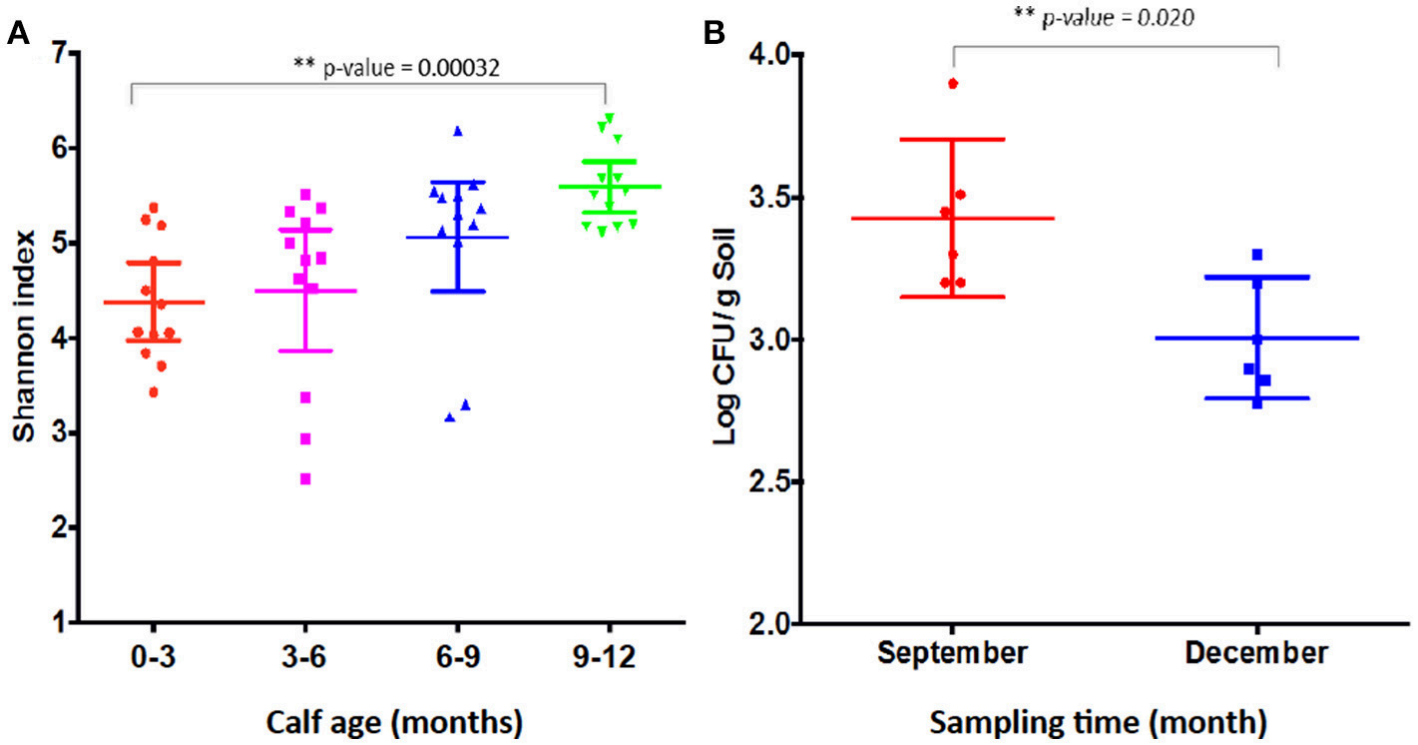
Shannon index and resistant bacterial concentration shed by calves. Shannon index of fecal samples from calves at different sampling times **(A)** and the concentration of resistant bacteria (Log CFU/g soil) **(B)** are presented. **(A)** Shows the Shannon index of older calves at 12 months of age in December sampling is significantly higher (*P-value* = 0.00032) than calves of 0–3 months of age samples in March at the same farm. The Shannon index was measured from the metagenomic analysis of the fecal samples collected from calves at four sampling periods. Each symbol represents individual fecal sample. **(B)** Shows the concentration of CRB in soil in September is significantly higher (*P*-value = 0.02) compared to December. The concentration was log transformed before the Students two-tailed *t*-test was applied to see the difference in the means of CRB among the 12 different soil samples. Each symbol represents individual soil sample.

Shannon index has been used to measure microbial diversity (Lozupone and Knight, 2008) and, as shown in Figure 8A, the diversity of microbiota increased as the calves age, which is consistent with the previous finding that the Shannon index increased as calves age (Mir et al., 2016a). Therefore, the lower prevalence of CRB at age 9–12 month might be associated with increased microbiota diversity in the gastrointestinal tract. However, as shown in Figure 2B, when we analyzed individual animals, the average CRB concentration in animals (*n* = 12) colonized with CRB was significantly higher compared to the younger animals, suggesting that the increased microbial diversity at age 9–12 may not entirely explain the phenomenon of the CRB colonization. In order to determine factors that may affect the prevalence of CRB in calves, we compared the concentration of CRB in soil samples in September, matching with the age of 6–9 month calves and December, matching with the age of 9–12 months. The prevalence of CRB in soil samples was significantly higher in September (10%) vs. the December (1%) sampling period. Also, as shown in Figure 8B, the concentration of CRB per gram of soil was significantly lower in December (*P* = 0.02). The lower prevalence and concentration of CRB in soil in December may have led to the lower colonization of calves at low temperature, suggesting the prevalence of CRB in calves is directly affected by environmental factors including temperature.

## Discussion

In this study a cohort of beef calves with no history of prophylactic or therapeutic cephalosporin antibiotic use was followed for1 year to investigate the phenomenon of naturally occurring antimicrobial resistance. The high prevalence (92%) of CRB during the early stages of life identified in this herd indicates that antimicrobial resistance in beef cattle can originate independently from antibiotic usage. Thus, not only the therapeutic or non-therapeutic use of antibiotics in food animals is contributing antibiotic resistance (Alekshun and Levy, 2007; Aminov and Mackie, 2007), but also the occurrence of antibiotic resistance in cattle is originated in the environment (Davies and Davies, 2010; Forsberg et al., 2012; Berendonk et al., 2015).

Since ESBLs confer resistance to critically important cephalosporin drugs (Mollenkopf et al., 2012) and ESBL genes could potentially be transferred between animals and humans (Tamang et al., 2013), the CRB isolated during this study carry a high level of public health significance. Not only were the ESBL gene sequences almost identical to those found on bacterial plasmids isolated from bacteria in patients hospitalized with antibiotic resistant infections (Enoch et al., 2012; Seiffert et al., 2013), but also the predominant species found in this study, *E. coli*, is often the causative bacterium of such infections (Burke et al., 2012). Moreover, all of the isolates subjected to antibiotic susceptibility tests were multi-drug resistant, with some isolates resistant toward up to nine different compounds. The higher prevalence of multi-drug resistant bacteria among young beef calves is alarming because these resistant isolates and genes can be spread to other bacteria including human pathogens by horizontal transmission of resistance genes (Sørensen et al., 2005; Doi et al., 2012), consumption of contaminated of beef (Hoyle et al., 2006) or accidental infection by agricultural workers (Marshall and Levy, 2011).

Though the reasons for the change in the proportion of *bla*CTX-M variants detected in this cohort of cattle remains uncertain, it could indicate that the ESBL genes are evolving in the commensal microbiota within the calf intestine or new plasmids are acquired from the environment. Given that the microbial community structure has been found to affect the antibiotic resistance in soil (Forsberg et al., 2014) and the calves in this study both gained and lost the presence of CRB, the evolution of plasmids in the soil microbiome and acquisition through grazing represents a plausible explanation.

We demonstrated the role of microbiota in the dynamics of cefotaxime resistance and our results indicate the difference in microbiota in cefotaxime resistant samples compared to the susceptible samples (Table 2). We also observed that different taxa were predominant at different sampling time points (Table 3) indicating that the microbiota is dynamic and develop as the animals grow or changes with the sampling times. Though it remains uncertain as to what extent the changes in gut microbiota affected the prevalence of CRB in these calves, it is possible that animals with a higher diversity contained more native bacterial populations in the gastrointestinal tract that made colonization of the surface epithelium by CRB ingested during grazing more difficult. The changes in the intestinal microbiota and the relationships between the commensal organisms present in fecal samples and the isolation of CRB require further research.

In addition, the calves in this study were weaned in August right after the third fecal sampling and left on the pasture with the diet consisting of grasses on the pasture without milk. Although we cannot exclude the possibility that weaning might have affected the prevalence of cefotaxime resistance because the diet is directly linked to the intestinal microbiota in animals (Schwab et al., 2011; Carmody et al., 2015), it is very unlikely. Because all calves grazed same grasses on the same pasture but 12 animals carried significantly high concentration of CRB while other did not carry any CRB. In contrast, we hypothesize that the prevalence of CRB in the gastrointestinal tract is more likely associated with the concentration of bacteria in soil rather than the maturity of microbiota in calves. This would be supported by the observation that the prevalence of CRB was low in calves in December, where lower concentration of CRBs in soil as a result of lower environmental temperatures that could reduce animal exposure. Indeed, lower concentrations of CRB were measured in December compared to September, which has warmer temperatures and higher concentrations of CRB in the soil (Figure 8B). Future studies should include a comparison of soil and cattle concentrations of CRB to examine the association between the prevalence of CRB in calves and environmental factors such as temperature.

During this cohort study, over 90% of the calves without any previous exposure to prophylactic or therapeutic antibiotics were colonized by CRB during the first year of life. Even though the exact origins of the genes responsible for antibiotic resistance found on this farm remain uncertain, the fact that these cattle have never been given antibiotics, nor has cefotaxime ever been used in animal husbandry, suggests that these genes were acquired in the environment. In that regard, it is impossible to determine if the acquisition of such genes by grazing food animals could have happened through evolution of the microbiota in the soil on this farm or that they were introduced into the soil via vectors (contaminated bird droppings or municipal waste water effluents). Regardless of origin, the beef cattle in this study represent a potential source of human exposure to ARMs, which contain genetic elements that can be transferred to pathogenic and commensal bacteria and may result in potentially resistant bacterial infections in humans.

## Author contributions

RM and KJ: designed the study; RM, TW, AK, LT, JD, ME, and KJ: collected and analyzed data; RM, TW, and AK: drafted manuscript; RM, TW, AK, LT, JD, ME, and KJ: finalized this manuscript.

### Conflict of interest statement

The authors declare that the research was conducted in the absence of any commercial or financial relationships that could be construed as a potential conflict of interest.

## Supplementary material

Figure S1 Antibiotic susceptibility of the isolates.
