# Supplementary figures and images for: Colonization Dynamics of Cefotaxime Resistant Bacteria in Beef Cattle Raised Without Cephalosporin Antibiotics
Source: Front Microbiol. 2018 Mar 21;9:500. doi: 10.3389/fmicb.2018.00500 (PMC5871660; doi:10.3389/fmicb.2018.00500)

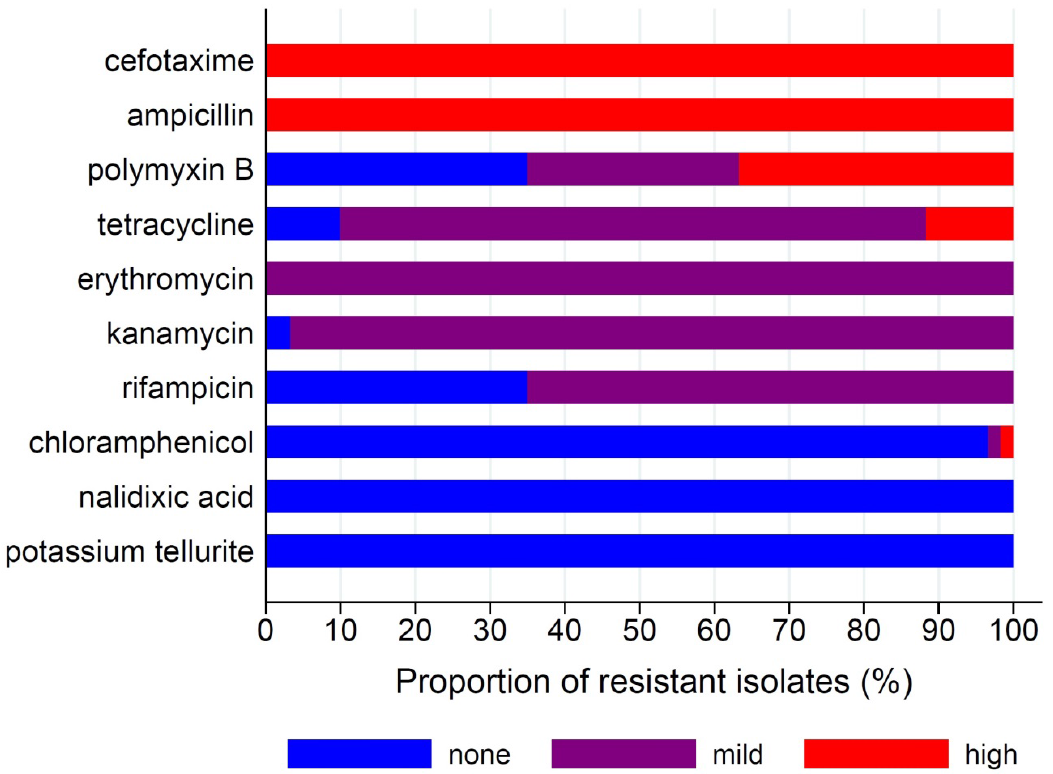

Supplement: Figure S1 — Antibiotic susceptibility of the isolates. [file Image1.TIF]
